# Perspectives on Human Cingulate Cortex Functional Organization From Cortical Electrical Stimulation Mapping

**DOI:** 10.1002/cns.71191

**Published:** 2026-09-28

**Authors:** Yun Zhuang, Haoyu Wang

**Affiliations:** ^1^ Ningbo Rehabilitation Hospital, School of Rehabilitation Medicine Wenzhou Medical University Zhejiang China

1

We read with great interest the recent article titled “Function of the Human Cingulate Cortex: A Brainnetome Atlas‐Based Study via Cortical Electrical Stimulation in Patients with Epilepsy” by Deng and colleagues [1], in which the authors established a comprehensive functional atlas of the human cingulate cortex using cortical electrical stimulation (CES) in 234 patients by integrating 1141 stimulation sites with the Brainnetome Atlas. The study provides one of the largest systematic characterizations of cingulate functions to date and represents a valuable resource for both cognitive neuroscience and functional neurosurgery. We congratulate the authors on this important contribution. We would like to offer several considerations that may further strengthen future investigations in this field.

A first consideration concerns the developmental trajectory of cingulate functional organization. The authors noted that their cohort included patients with diverse pathologies and a broad age range, and suggested that future studies in more homogeneous adult cohorts may help refine the functional organization of the cingulate cortex [1]. However, we suggest that the wide age distribution of the present cohort (mean age 20.0 ± 10.2 years) may also provide an opportunity to investigate developmental changes in cingulate functional organization. The cingulate cortex undergoes protracted structural and functional maturation throughout adolescence and early adulthood, with developmental changes in cortical morphology [2] and functional connectivity [3]. Moreover, structural changes in the cingulate cortex continue throughout adulthood, with individual subregions showing distinct trajectories of age‐related volume decline [4]. Given that the present cohort spans from late childhood to mid‐adulthood, an initial comparison between adolescent (< 18 years) and adult (≥ 18 years) groups could reveal age‐related changes in subregional distribution or stimulation thresholds. If sample sizes permit, modeling age as a continuous variable would further capture the progressive nature of cortical maturation. Given the sampling constraints inherent to clinical electrode placement, integration with functional magnetic resonance imaging (fMRI) developmental studies would further strengthen such analyses.

A second consideration concerns the interpretation of variability in cingulate stimulation responses. The authors adopted a group‐level analytical approach by aggregating stimulation responses across 1141 sites from 234 patients. As reviewed by Borchers et al. [5], CES responses reflect complex interactions between local excitability, axonal activation, and network‐level propagation, introducing potential sources of variability that may not directly correspond to functional specialization. The large sample size of the present study is therefore a major strength that enables more reliable characterization of population‐level functional organization. We suggest that quantifying cross‐subject consistency of responsive sites could provide an informative assessment of robustness. For each function‐by‐subregion combination, metrics that capture the proportion of sampled patients contributing to the observed responses would help distinguish functional associations that are consistent across individuals from those driven by a small subset of patients.

A third consideration concerns the spatial organization of distinct functional domains within the cingulate cortex. The present study provides a systematic mapping of stimulation‐induced responses across seven subregions and, consistent with previous work [6, 7, 8], supports the view that the cingulate cortex participates in multiple processes, including cognitive, emotional, and interoceptive representation. An important question arising from this functional diversity is whether different domains are spatially segregated or organized along continuous gradients. Comparing pairwise spatial distances among sites of different functional categories could provide a quantitative approach to addressing this question. Given the variability in electrode spatial sampling, integration with functional imaging data would strengthen such analyses. Moreover, the 67 sites that elicited more than one response type represent potential regions of functional convergence and warrant further investigation.

In summary, the Deng et al. [1] study represents a significant contribution to cingulate functional neuroanatomy. We respectfully suggest that developmental subgroup analysis, interindividual variability assessment, and connectivity‐informed interpretation will enhance the precision and interpretive value of future cingulate functional atlases. We thank the authors for their contribution and hope these perspectives may inform their ongoing investigations.

## Ethics Statement

The authors have nothing to report.

## Conflicts of Interest

The authors declare no conflicts of interest.

## Data Availability

Data sharing not applicable to this article as no datasets were generated or analysed during the current study.
